# Supplementary material for: A mouse model of occult intestinal colonization demonstrating antibiotic-induced outgrowth of carbapenem-resistant Enterobacteriaceae
Source: Microbiome. 2022 Mar 10;10:43. doi: 10.1186/s40168-021-01207-6 (PMC8908617; doi:10.1186/s40168-021-01207-6)
Supplement: Supplementary file 2 — Additional file 1. a Table showing minimum inhibitory concentration (MIC) of antibiotics on wild-type KPNIH1 derivative (MKP103), evolved strain (xylR-L4) and transposon strain (xylR-Tn1). ND, not determined. b Mice were orally gavaged with 105 CFU of K. pneumoniae at day 0. Antibiotic cocktail was given from day 7 to day 21. CFU of K. pneumoniae in the stated samples at day 21. N=6. c Table showing antibiotics used in study, their drug class, mechanisms of actions and target bacteria. [file 40168_2021_1207_MOESM2_ESM.pdf]

Additional file 1

a

	Antibiotic	MKP103	xylR-L4	xylR-Tn1
1	Ampicillin	>256	>256	>256
2	Vancomycin	>256	>256	>256
3	Neomycin	256	ND	ND
4	Metronidazole	>256	ND	ND
5	Ciprofloxacin	>32	ND	ND
6	Azithromycin	>256	ND	ND
7	Rifaximin	>256	ND	ND

ND = not determined

b

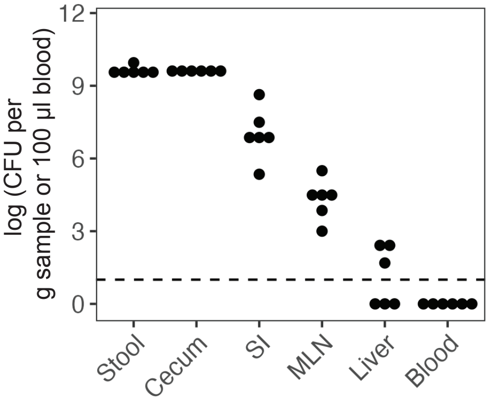

c

	Antibiotic	Drug class	Mechanism	Target
1	Cocktail (AMNV)	See Below	See Below	See Below
2	Ampicillin (A)	beta-lactam, penicillin	Inhibits cell wall synthesis	Many gram-positive and some gram-negative bacteria
3	Metronidazole (M)	Nitroimidazole	Destabilizes DNA	Anaerobes
4	Neomycin (N)	Aminoglycoside	Inhibits protein synthesis	Gram-positive and -negative bacteria
5	Vancomycin (V)	Glycopeptide	Inhibits cell wall synthesis	Gram-positive bacteria
6	Ciprofloxacin	Fluoroquinolone	Inhibits DNA gyrase	Some gram-positive and many gram-negative bacteria
7	Azithromycin	Macrolide	Inhibits protein synthesis	Many gram-positive and some gram-negative bacteria
8	Rifaximin	Rifamycins	Inhibits RNA polymerase	Gram-positive and -negative bacteria
