# Supplementary figures and images for: A mouse model of occult intestinal colonization demonstrating antibiotic-induced outgrowth of carbapenem-resistant Enterobacteriaceae
Source: Microbiome. 2022 Mar 10;10:43. doi: 10.1186/s40168-021-01207-6 (PMC8908617; doi:10.1186/s40168-021-01207-6)

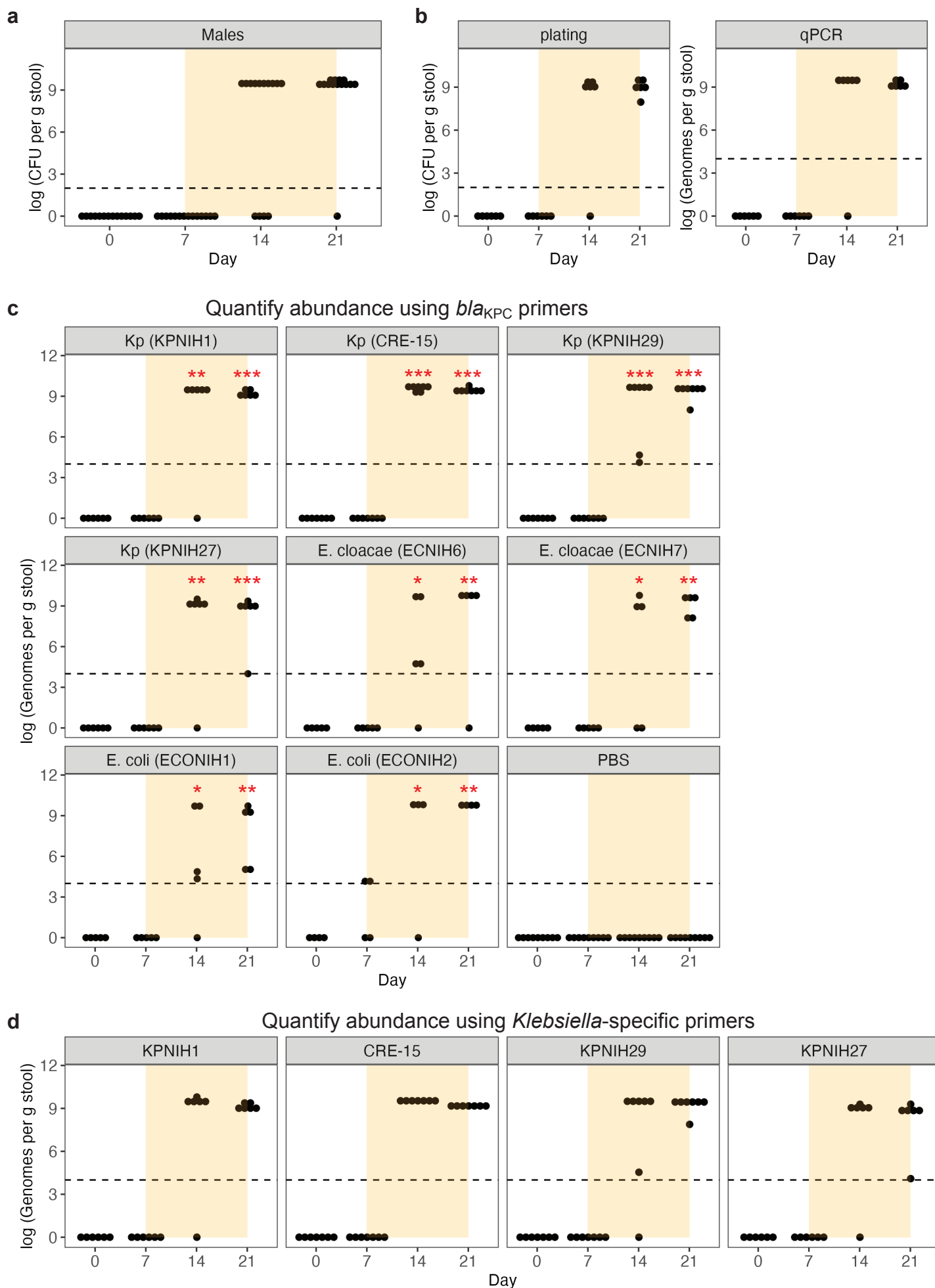

Supplement: Supplementary file 3 — Additional file 2. a Male mice were orally gavaged with 105 CFU of K. pneumoniae at day 0. Ampicillin was given from day 7 to day 21. CFU of K. pneumoniae in stool at day 0, 7, 14, and 21. N=14. The dotted line marks the limit of detection. b Mice were orally gavaged with 105 CFU of KPNIH1 K. pneumoniae at day 0. Ampicillin was given from day 7 to day 21. Levels of K. pneumoniae in stool at day 0, 7, 14, and 21 were determined by plating on antibiotic cocktail agar or qPCR using primers at blaKPC gene. N=6. c KPC-producing strains of K. pneumoniae, E. cloacae and E.coli occultly colonize the gut until robust outgrowth by ampicillin. Mice were orally gavaged with 105 CFU of each strain or PBS at day 0. Ampicillin was given from day 7 to day 21. Levels of Enterobacteriaceae in stool at the stated time-points. Measurement was done by qPCR using primers at blaKPC gene. N=4-9. d Levels of K. pneumoniae were also measured using primers that bind to a chromosomal gene at the Klebsiella capsular polysaccharide synthesis gene cluster. For (c), statistics was performed using Fisher’s exact test with Hommel correction comparing each strain to PBS control on each time-point. *p-value < 0.05, **p-value < 0.01, ***p-value < 0.001. [file 40168_2021_1207_MOESM3_ESM.pdf]

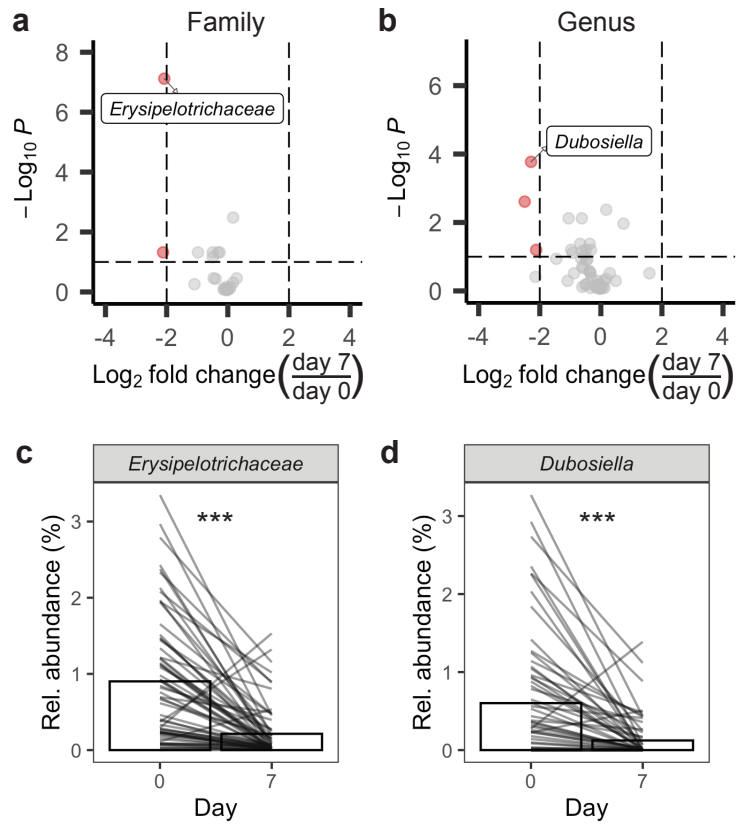

Supplement: Supplementary file 4 — Additional file 3. a, b Volcano plots showing adjusted p-value versus fold change of relative abundances of taxa at (a) family level and (b) genus level between day 0 and day 7. c, d Relative abundances of (c) Erysipelotrichaceae and (d) component genus Dubosiella. Bars show mean abundance. N=64. For (a-d), p-value is calculated by paired Wilcoxon signed-rank test with Benjamini-Hochberg correction. ***p-value < 0.001. [file 40168_2021_1207_MOESM4_ESM.pdf]
