# Supplementary material for: A mouse model of occult intestinal colonization demonstrating antibiotic-induced outgrowth of carbapenem-resistant Enterobacteriaceae
Source: Microbiome. 2022 Mar 10;10:43. doi: 10.1186/s40168-021-01207-6 (PMC8908617; doi:10.1186/s40168-021-01207-6)
Supplement: Supplementary file 5 — Additional file 4. a Percentage of reads with a Kraken2 taxonomic classification using standard RefSeq or GTDB databases. Error bars denote SEM. b Relative abundance of the only significant genus Akkermansia between susceptible and nonsusceptible microbiota. c Top 5 species that show a trend between susceptible and nonsusceptible microbiota. d Percentage of reads with an eggNOG functional annotation. For (a), p-value is calculated by a two-tailed t-test. For (b) and (c), p-value is calculated using the linear model in MaAsLin2 and adjusted by Benjamini-Hochberg. *p-value < 0.05, ***p-value < 0.001. [file 40168_2021_1207_MOESM5_ESM.pdf]

Additional file 4

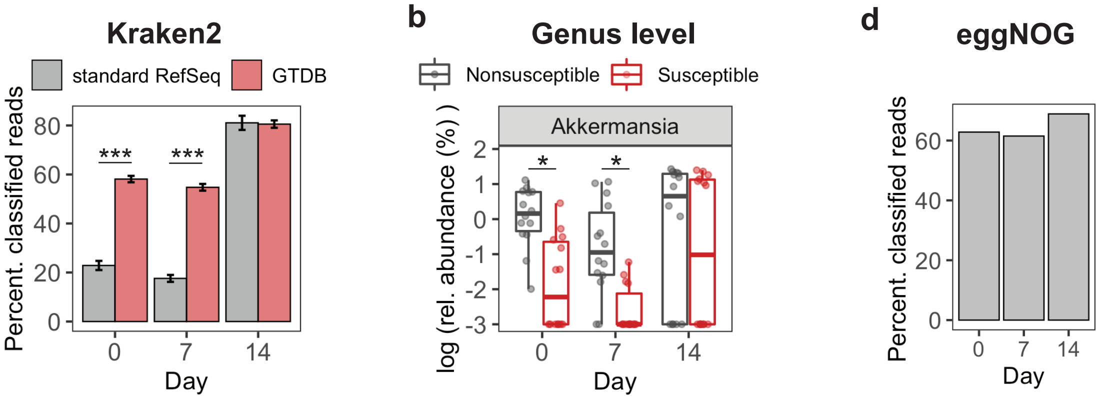

c

	day 0		day 7	
**Species level**	**adjusted pval**	**enriched**	**adjusted pval**	**enriched**
Akkermansia muciniphila A	0.122	nonsuscep	0.156	nonsuscep
Bacteroides ovatus	0.122	nonsuscep	0.156	nonsuscep
Duncaniella sp001701225	0.122	suscep	> 0.2	suscep
Alistipes sp003979135	0.122	suscep	> 0.2	suscep
Akkermansia muciniphila	0.122	nonsuscep	> 0.2	nonsuscep
