# Supplementary material for: A mouse model of occult intestinal colonization demonstrating antibiotic-induced outgrowth of carbapenem-resistant Enterobacteriaceae
Source: Microbiome. 2022 Mar 10;10:43. doi: 10.1186/s40168-021-01207-6 (PMC8908617; doi:10.1186/s40168-021-01207-6)
Supplement: Supplementary file 6 — Additional file 5. a Table showing mutations in evolved strains. A representative strain is displayed per lineage. b Overall outgrowth statistics of the stated antibiotics from all the mice used in the project. [file 40168_2021_1207_MOESM6_ESM.pdf]

Additional file 5

a

Evolved lineage	Mutation (SNP/ deletion)	Gene	Gene product
L1	missense_variant c.163C>T p.His55Tyr	KPNIH1_02290	30S ribosomal protein S6
	conservative_inframe_deletion c.399_404delCGGCGA p.Gly134_Asp135del	KPNIH1_18070	porin OmpC
	missense_variant c.765G>T p.Met255Ile	KPNIH1_22140	S-adenosylmethionine synthetase
	missense_variant c.718C>T p.Arg240Cys	KPNIH1_25345	XylR family transcriptional regulator
L2	missense_variant c.199A>G p.Thr67Ala	KPNIH1_12760	DeoR family transcriptional regulator
	missense_variant c.448A>C p.Thr150Pro	KPNIH1_24015	multidrug transporter
	missense_variant c.796A>T p.Ile266Phe	KPNIH1_25345	XylR family transcriptional regulator
	stop_gained c.543G>A p.Trp181*	KPNIH1_26320	phosphate ABC transporter substrate-binding protein
L3	conservative_inframe_deletion c.399_404delCGGCGA p.Gly134_Asp135del	KPNIH1_18070	porin OmpC
	missense_variant c.487G>A p.Ala163Thr	KPNIH1_25345	XylR family transcriptional regulator
L4	missense_variant c.839G>A p.Arg280Gln	KPNIH1_25345	XylR family transcriptional regulator
L5	missense_variant c.53T>G p.Ile18Ser	KPNIH1_03765	two-component response regulator DpiA
	conservative_inframe_deletion c.399_404delCGGCGA p.Gly134_Asp135del	KPNIH1_18070	porin OmpC
	missense_variant c.235C>T p.Pro79Ser	KPNIH1_25345	XylR family transcriptional regulator
L6	missense_variant c.179G>A p.Arg60His	KPNIH1_17610	LacI family transcriptional regulator
	conservative_inframe_deletion c.399_404delCGGCGA p.Gly134_Asp135del	KPNIH1_18070	porin OmpC

b

Antibiotic	No. of mice tested	Outgrowth (%) at day 21
cocktail	45	91.1
amp	96	99.0
van	141	50.4
azm	48	29.2
Total	330	
