# Supplementary material for: A mouse model of occult intestinal colonization demonstrating antibiotic-induced outgrowth of carbapenem-resistant Enterobacteriaceae
Source: Microbiome. 2022 Mar 10;10:43. doi: 10.1186/s40168-021-01207-6 (PMC8908617; doi:10.1186/s40168-021-01207-6)
Supplement: Supplementary file 7 — Additional file 6. a Primer sequences. b Bacterial strains. [file 40168_2021_1207_MOESM7_ESM.pdf]

a

**Allele**	**Forward primer (5' to 3')**	**Reverse primer (5' to 3')**	**Notes**
blaKPC	CCATCCGTTACGGCAAAAAT	TTATCACTGTATTGCACGGCG	For figure 2e and Additional file 2b/2c
xylR-L4	ACGCTCCACCGACTACCA	CCCCGACCTCCTCTTTGAAG	For figure 5f
xylR-WT (control for L4)	ACGCTCCACCGACTACCG	CCCCGACCTCCTCTTTGAAG	For figure 5f
xylR-Tn1	GGTCCACTATATCGCCACCG	GAACACCCGAGAAAATTCATCG	For figure 5e. Use PCR elongation time = 20 sec
xylR-WT (control for Tn1)	GGTCCACTATATCGCCACCG	GGCATACTCGCGCTCTACC	For figure 5e. Use PCR elongation time = 20 sec
KPNIH1_17390	GAATACCTTTCGCCTTGATGC	GTGGTGGGTGTGTATCGC	Klebsiella-specific. For Additional file 2d

b

**Bacterial strains**	**Description**	**Source**
MKP103	K. pneumoniae, ST258, blaKPC-, derived from KPNIH1 strain	(Ramage et al. 2017)
KPNIH1	K. pneumoniae patient isolate, ST258, blaKPC+	(Conlan et al. 2014)
KPNIH29	K. pneumoniae patient isolate, ST1518, blaKPC+	(Conlan et al. 2014)
KPNIH27	K. pneumoniae patient isolate, ST34, blaKPC+	(Conlan et al. 2014)
CRE-15	K. pneumoniae patient isolate, ST45, blaKPC+	NCBI accession: SAMN04014967
ECONIH1	E. coli patient isolate, ST648, blaKPC+	(Conlan et al. 2014)
ECONIH2	E. coli patient isolate, ST127, blaKPC+	(Hardiman et al. 2016)
ECNIH6	E. cloacae patient isolate, ST191, blaKPC+	(Chen et al. 2017)
ECNIH7	E. cloacae patient isolate, ST53, blaKPC+	(Chen et al. 2017)
xylR-L1	K. pneumoniae, from evolution lineage 1, contains a missense SNP at xylR gene	This study
xylR-L2	K. pneumoniae, from evolution lineage 2, contains a missense SNP at xylR gene	This study
xylR-L3	K. pneumoniae, from evolution lineage 3, contains a missense SNP at xylR gene	This study
xylR-L4	K. pneumoniae, from evolution lineage 4, contains a missense SNP at xylR gene	This study
xylR-L5	K. pneumoniae, from evolution lineage 5, contains a missense SNP at xylR gene	This study
xylR-Tn1	K. pneumoniae, loss-of-function mutant of xylR gene, from MKP103 transposon library #KP10497	(Ramage et al. 2017)
xylR-Tn2	K. pneumoniae, loss-of-function mutant of xylR gene, from MKP103 transposon library #KP10499	(Ramage et al. 2017)
